# A monocarboxylate transporter-dependent mechanism confers resistance to exercise-induced fatigue in a high-altitude hypoxic environment

**DOI:** 10.1038/s41598-023-30093-1

**Published:** 2023-02-20

**Authors:** Chen Gao, Binni Yang, Yurong Li, Wenjuan Pei

**Affiliations:** Department of General Practice, The 940Th Hospital of Joint Logistics Support Force of Chinese People’s Liberation Army, BinHe South Road, No.333, Lanzhou, 730050 Gansu China

**Keywords:** Neuroscience, Physiology, Medical research, Molecular medicine

## Abstract

The body is more prone to fatigue in a high-altitude hypoxic environment, in which fatigue occurs in both peripheral muscles and the central nervous system (CNS). The key factor determining the latter is the imbalance in brain energy metabolism. During strenuous exercise, lactate released from astrocytes is taken up by neurons via monocarboxylate transporters (MCTs) as a substrate for energy metabolism. The present study investigated the correlations among the adaptability to exercise-induced fatigue, brain lactate metabolism and neuronal hypoxia injury in a high-altitude hypoxic environment. Rats were subjected to exhaustive incremental load treadmill exercise under either normal pressure and normoxic conditions or simulated high-altitude, low-pressure and hypoxic conditions, with subsequent evaluation of the average exhaustive time as well as the expression of MCT2 and MCT4 in the cerebral motor cortex, the average neuronal density in the hippocampus, and the brain lactate content. The results illustrate that the average exhaustive time, neuronal density, MCT expression and brain lactate content were positively correlated with the altitude acclimatization time. These findings demonstrate that an MCT-dependent mechanism is involved in the adaptability of the body to central fatigue and provide a potential basis for medical intervention for exercise-induced fatigue in a high-altitude hypoxic environment.

## Introduction

Fatigue refers to the decreased capacity or complete inability of an organism, organ, or part to function normally because of excessive stimulation or prolonged exertion^1^; it is a feeling of tiredness. Physical fatigue is the transient inability of muscles to maintain optimal physical performance and is made more severe by intense physical exercise^2–4^. Physical fatigue can be caused by a lack of energy in the muscle, by a decrease in the efficiency of the neuromuscular junction or by a reduction in the drive originating from the central nervous system^5^, which can be restored after proper rest time and adjustment. It is an extremely complex body comprehensive reaction process. Critical power (CP) represents the boundary between the intensity domains of heavy and severe exercise^6^. It represents an important parameter of aerobic function and is the greatest average effort that can be sustained for a period of time without fatigue^7^. Fatigue is more likely to occur at high altitudes and is usually attributed to low oxygen levels^8–11^. However, millions of people live and work at high altitudes, and many of them are able to adjust successfully to the hypoxic environment of very high altitudes, which is called altitude acclimatization and is a slow physiological adaptation resulting from prolonged exposure to significantly reduced atmospheric pressure^12^. Discovery of the mechanisms responsible for human acclimatization to hypoxia could lead to new ways to improve acclimatization and retention.

Fatigue is considered to be an interactive effect resulting from both peripheral muscles and CNS factors. The key factor determining the latter is the imbalance in brain energy metabolism, which makes it difficult to maintain sufficient peripheral nerve impulses to the muscles continuously^13,14^. The mechanism is associated with the decrease in brain electrical activity caused by excessive oxygen consumption during exhaustive exercise and the feedback inhibition of activity in the CNS and anterior motoneurons^15–17^.

Under strenuous exercise or a hypoxic environment, the body maintains the balance of energy metabolism through anaerobic glycolysis of carbohydrates such as glycogen, and lactate is the metabolite of this process^18–20^. The rapid decline in body exercise capacity after rush entry into altitude is related to the accumulation of lactate, which triggers a series of pathological responses to damage neurons by reducing the pH under the hypoxic high-altitude environment^21,22^. However, long-term altitude life leads to altitude acclimatization, and body physiological functions such as antifatigue ability are recovered to some extent. The best strategy to enhance the exercise capacity of the body at high altitude is to search for measures that are effective in improving the acclimation ability and shortening the acclimation time^23^. However, the molecular mechanism of altitude acclimatization in the CNS is not known. In recent years, studies have proven that lactate can be used as a source of energy metabolism under certain circumstances^18,24,25^. During sustained exercise, the turnover and oxidation rates of lactate exceed those of glucose. The diversion of lactate carbon to oxidation during exercise and recovery represents an irreversible loss of gluconeogenic precursors because the processes of protein proteolysis and gluconeogenesis from amino acids are insufficient to achieve complete glycogen restitution after exhausting exercise^26^. The “astrocyte-neuron lactate shuttle hypothesis”^27,28^ indicates that under extreme exercise or hypoxic conditions, lactate released from glial cells by anaerobic metabolism is taken up by neurons and used as an energy substrate^29,30^ and that lactate is oxidized preferentially over glucose for neuronal utilization^31^. The body lactate exchange carriers are monocarboxylate transporters (MCTs), which are widely distributed on tissue cell membranes and can be divided into 14 subtypes^32^. The central lactate shuttle is mainly achieved by a closed loop formed by MCT2 and MCT4 specifically expressed by neurons and astrocytes, respectively^33^. Therefore, we speculated that the mechanism of altitude acclimation in the CNS is related to the metabolism and utilization of lactate in the brain. To test this hypothesis, we designed a randomized controlled trial based on an animal model of altitude exercise-induced fatigue. The variation trend in the exercise capacity of rats was recorded under a simulated high-altitude hypoxic environment, with a subsequent evaluation of the expression of MCT2 and MCT4 in the cerebral motor cortex as well as neuronal hypoxia injury in the hippocampal CA1 subfield and the lactate content in the rat brain. The aim of this study was to explore the correlation between exercise-induced fatigue and brain lactate metabolism to provide a potential basis for medical intervention for exercise-induced fatigue in a high-altitude hypoxic environment.

## Results

### Exhaustive time of load exercise in rats (Fig. 1)

**Figure 1:**
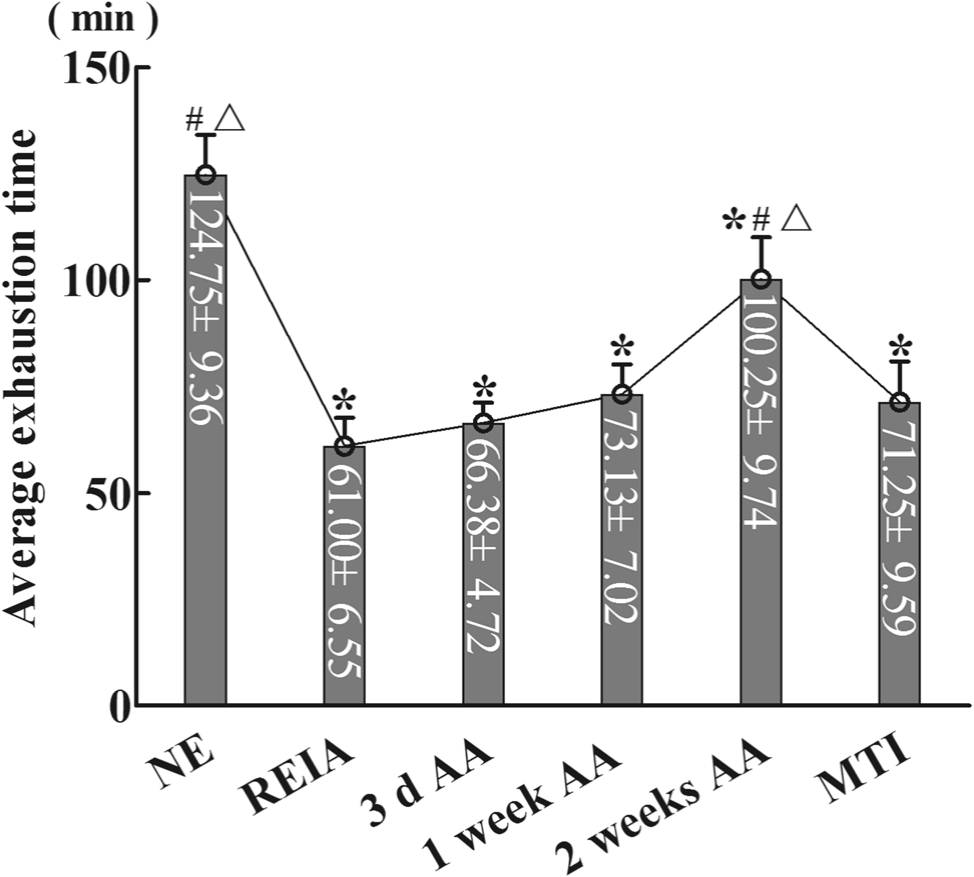
Average exhaustive time in minutes of load exercise of rats in each group. Data are presented as the mean ± SD. *P < 0.05 vs. the NE group, #P < 0.05 vs. the REIA group, △P < 0.05 vs. the MTI group (ANOVA with post hoc Bonferroni correction; n = 9 per group).

The average exhaustion times in the REIA group, 3 d AA group, 1 week AA group, 2 weeks AA group and MTI group were 61.00 ± 6.55 min, 66.38 ± 4.72 min, 73.13 ± 7.02 min, 100.25 ± 9.74 min and 71.25 ± 9.59 min, respectively, which were significantly lower than that in the NE group (124.75 ± 9.36 min) (*p* = 0.000). As the time of altitude acclimatization was prolonged, the average exhaustive time in the 2 weeks AA group significantly recovered to 100.25 ± 9.74 min, and there was a significant difference compared with that in the REIA group and MTI group (*p* = 0.000). After injection of 4-CIN, the exercise capacity of rats was significantly decreased. The average exhaustive time of the MTI group was not significantly different from that of the REIA group (*p* = 0.219). Therefore, the exhaustive time of rats in each group under simulated high-altitude, low-pressure and hypoxic conditions showed a sharp decline after entering altitude and then gradually increased with the prolongation of altitude acclimatization.

### Expression of MCT2 and MCT4 in the cerebral motor cortex of rats

**Figure 2:**
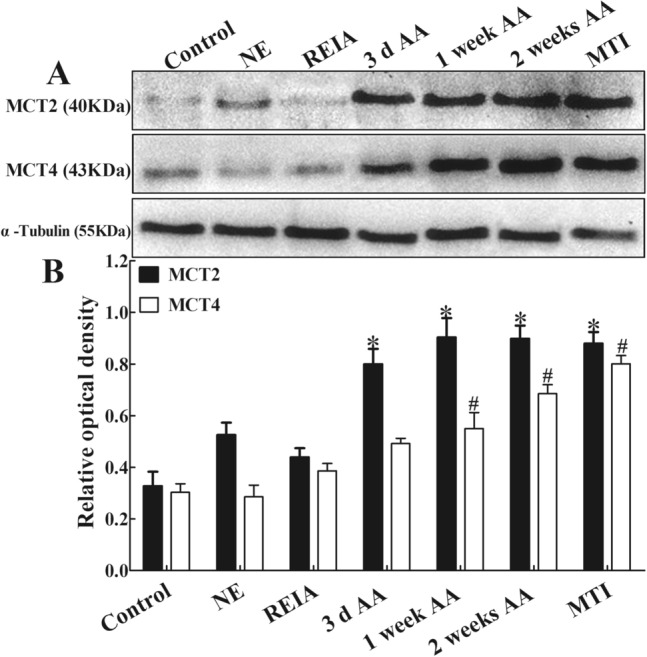
Western blot analysis of MCT2 and MCT4 in the cerebral motor cortex of rats in each group. (**A**) Representative cropped immunoblots showing a 40-kDa band corresponding to MCT2 protein and a 43-kDa band corresponding to MCT4 protein, with a-tubulin (55-kDa band) as a loading control. Original blots are presented in the Supplementary Figure original blots. (**B**) Results of the relative quantitative analysis. *P < 0.05 vs. the control group for MCT2; ^#^P < 0.05 vs. the control group for MCT4 (ANOVA with post hoc Bonferroni correction; n = 3 per group). The original images of full-length blots with membrane edges visible can be found in the Supplementary Information file.

The results of Western blotting showed that MCT2 and MCT4 proteins in the cerebral motor cortex of rats were visible as 40- and 43-kDa bands, respectively, in the immunoblot (Fig. 2A), and the results of the relative quantitative analysis are shown in Fig. 2B. MCT2 and MCT4 were expressed minimally and normally in the cerebral motor cortex of rats in the control group. Compared with the control group, there was no significant difference in the NE group and REIA group (*p* = 0.082, 0.303 for MCT2;* p* = 0.254, 0.436 for MCT4). After altitude acclimatization, the expression of MCT2 in the 3d AA group, 1 week AA group, 2 weeks AA group and MTI group increased earlier than that of MCT4 and was significantly higher than that in the control group by 144.3%, 176.1%, 174.6% and 168.8%, respectively (*p* = 0.000). The expression of MCT4 in the 1 week AA group, 2 weeks AA group and MTI group was significantly higher than that in the control group by 81.5%, 120.6% and 164.4%, respectively (*p* = 0.000).

**Figure 3:**
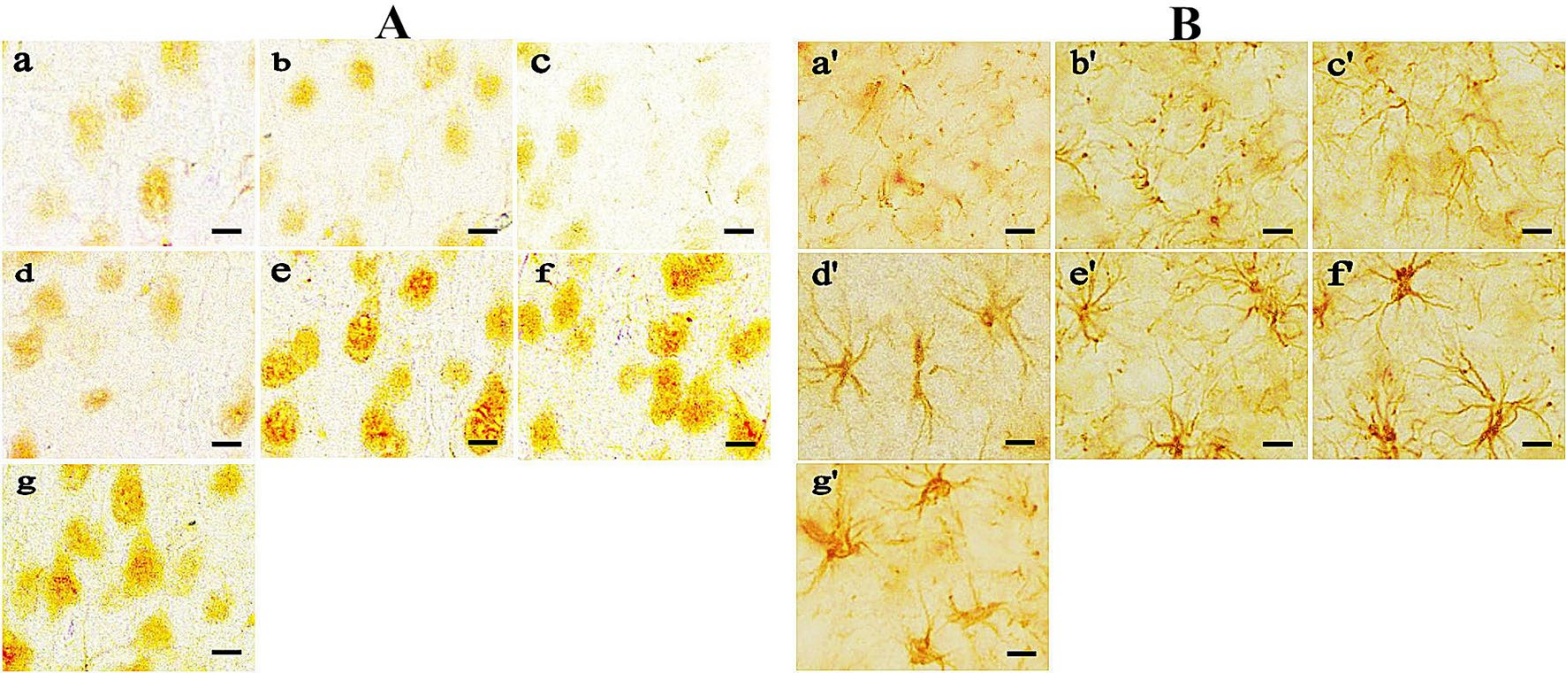
Immunohistochemical staining (3,3-diaminobenzidine (DAB) chromogen method) of MCT2 (**A**) and MCT4 (**B**) in the cerebral motor cortex of rats in each group. a,a’ control group, b,b’ NE group, c,c’ REIA group, d,d’ 3 d AA group, e,e’ 1 week AA group, f,f’ 2 weeks AA group, g,g’ MTI group. Scale bar = 25 μm.

Immunohistochemical staining methods (DAB method) were also used to detect the expression of MCT2 in neurons and MCT4 in astrocytes in the cerebral motor cortex of rats. As shown in Fig. 3, compared with the control group, the expression of MCT2 on neurons in the cerebral motor cortex of rats increased significantly and remained stable after 1 week of altitude acclimatization. The expression of MCT4 on astrocytes began to increase after 3 days of altitude acclimatization and remained stable after 2 weeks of altitude acclimatization. The variation trend of immunohistochemical detection was consistent with the western blotting results, which confirmed each other.

### Neuropathological evaluation of delayed neuronal death in the hippocampus of rats

**Figure 4:**
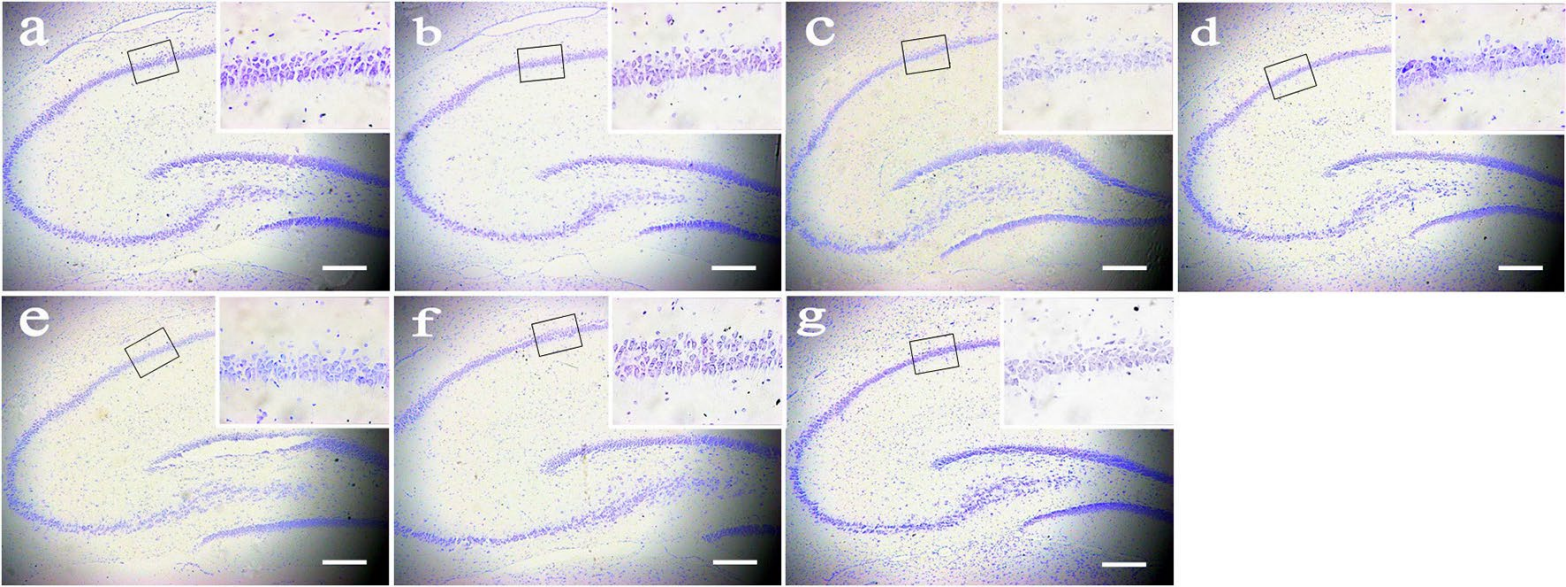
Nissl’s staining in the cerebral motor cortex of rats in each group. (**a**) control group, (**b**) NE group, (**c**) REIA group, (**d**) 3 d AA group, (**e**) 1 week AA group, (**f**) 2 weeks AA group, (**g**) MTI group. Scale bar = 100 μm.

Delayed neuronal death was evaluated by Nissl staining of brain sections in the hippocampal CA1 subfield of rats. The HG grades and mean ND values of rat brain neurons in each group are shown in Table 1. The mean ND values in the REIA group, 3-d AA group and MTI group were significantly lower than those in the control group (*p* = 0.000). The mean ND values in the 1 week AA group were still lower than those in the control group (*p* = 0.037), but there was no significant difference between the control group and the NE group or 2 weeks AA group (*p* = 0.399 for NE group, *p* = 0.132 for NE group). Representative Nissl staining of brain sections in the hippocampus of rats in each group is shown in Fig. 4. The cortical neurons in the control group, NE group, 1 week AA group and 2 weeks AA group were arranged regularly and evenly. The boundary between the cells was clear. The cell membrane and nucleus were intact, the nucleoli were clear, and the chromatin was evenly distributed. In the REIA group, 3d AA group and MTI group, the cortical neurons were arranged loosely and in a disorderly fashion, the boundary between cells was not clear, and the chromatin had gathered. In particular, the cytoskeleton and organelles were obviously destroyed, and some apoptotic or necrotic cells showed pyknosis and deep staining in the REIA group and 3 d AA group.

**Table 1:** The histological grade (HG) and pyramidal neuronal density (ND) of the cerebral motor cortex of rats in each group.

Group	Histological grade^a^				Neuronal density^b^
	0	I	II	III	
Control	3	0	0	0	135.88 ± 8.59
NE	2	1	0	0	123.88 ± 6.71
REIA	0	0	2	1	46.75 ± 8.65*
3 d AA	0	0	3	0	54.13 ± 11.33*
1 week AA	0	2	1	0	119.50 ± 6.99*
2 weeks AA	0	3	0	0	121.75 ± 16.00
MTI	0	1	2	0	63.50 ± 7.65*

*P < 0.05 vs. the control group. (ANOVA with post hoc Bonferroni correction; n = 3 per group.)

^a^Data indicate case numbers.

^b^Data are presented as the mean ± SD of cells/high power field (HPF).

### *Lactate content in the rat brain (Fig. **5**)*

**Figure 5:**
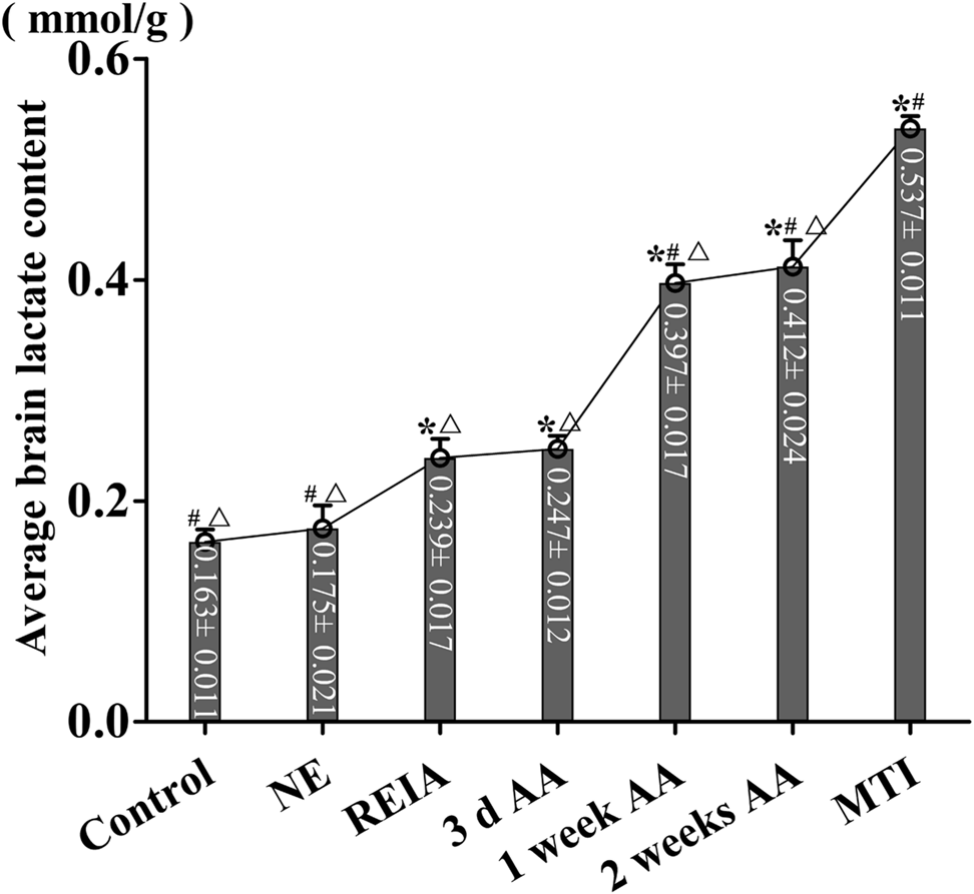
Average lactate content (in mmol/g) of rat brains in each group. Data are presented as the mean ± SD. *P < 0.05 vs. the NE group, #P < 0.05 vs. the REIA group, △P < 0.05 vs. the MTI group (ANOVA with post hoc Bonferroni correction; n = 3 per group).

The lactate contents of rat brains in the REIA group, 3 d AA group, 1 week AA group, 2 weeks AA group and MTI group were 0.239 ± 0.017 mmol/g, 0.247 ± 0.012 mmol/g, 0.397 ± 0.017 mmol/g, 0.412 ± 0.024 mmol/g and 0.537 ± 0.011 mmol/g, respectively, and were significantly higher than that in the NE group (0.175 ± 0.021 mmol/g) (*p* = 0.000) and showed a gradual upwards trend with the prolongation of altitude acclimatization. After 1 week of altitude acclimatization, the increasing trend of the lactate content in the rat brain was significantly accelerated, and the lactate content of the rat brain in the MTI group was the highest, which was significantly different from all of the other groups (*P* = 0.000).

## Discussion

The most significant and lasting effect of altitude hypoxia on the body is the reduction in exercise capacity. Studies have shown that the maximum exercise capacity of the human body at an altitude of 4500 m can decline to 50% of that in plain areas^23^. With the prolongation of the residence time in high-altitude areas, the body becomes acclimated to high-altitude environments, and the exercise capacity is gradually recovered^34^. As one of the core contents of altitude sports medicine research, altitude acclimatization has become a focus in altitude physiology. Previous studies have shown that central lactate accumulation can cause neuronal injury and can also be used by neurons as an energy substrate in exhaustive exercise or under anaerobic conditions^27,35^. Lactate transport between cells is dependent on MCTs, which are divided into 14 subtypes^33^, among which MCT2 and MCT4 are essential for lactate transport in the CNS during exercise^36^. This study indicates that the whole link between lactate excretion and uptake in the rat brain shows positive changes in adaptation to exercise-induced fatigue under a simulated high-altitude hypoxic environment. Therefore, it is speculated that the altitude acclimatization phenomenon is closely related to the balance between neuronal injury caused by central lactate accumulation and the oxidative energy supply.

Previous studies have shown that the “shuttling” of oxidizable substrate in the form of lactate from areas with a high glycogenolytic rate to areas with high cellular respiration through the interstitium and vasculature appears to represent an important means by which substrate is distributed, metabolic “waste” is removed, and the functions of various tissues are coordinated during exercise^26^. 4-CIN is a specific mitochondrial covalent transport inhibitor of lactate and pyruvate^37–40^. Different subtypes of MCTs have different sensitivities to 4-CIN, which is a competitive inhibitor. 4-CIN has the most obvious inhibitory effect on the transport function of MCT2; however, the sensitivity of MCT4 is lower^41,42^. The “astrocyte-neuron lactate shuttle hypothesis”^27,28^ indicates that lactate is released from glial cells via MCT4 and then is transported into neurons via MCT2 as a substrate for energy metabolism. Regardless of which link in this closed energy metabolic loop is effectively inhibited by competitive inhibition, the entire process can be effectively blocked. Therefore, the effect of regulating lactate transportation on exercise-induced fatigue at altitude was studied by intraperitoneal injection of 4-CIN in rats as an interfering factor. The results showed that the average exhaustive time of the rats was significantly shortened after rush entry into altitude, and the exercise capacity gradually recovered with the extension of altitude acclimatization. The expression levels of MCT2 and MCT4 in the cerebral motor cortex of rats were positively correlated with the altitude acclimatization time. Western blotting and immunohistochemical detection showed the same variation trend, thereby confirming each other. The recovery of exercise capacity after altitude acclimatization can be rapidly blocked by using 4–CIN as an interference factor. From the perspective of 4-CIN as a competitive inhibitor of lactate transport, we believe that the blockage of exercise capacity recovery is mainly due to the effective inhibition of the lactate transport function of MCT2 in the energy metabolism loop, which then affects the energy metabolism of neurons.

Hypoxia regulates the expression of a subset of astrocyte–neuron-specific transporters, including glucose and glutamate transporters as well as MCTs^43^. Previous studies have shown that the expression of MCT4 is increased in astrocyte–neuron cocultures during hypoxia, and neurons use lactate produced by glial cells as an energy substrate to resist oxygen and glucose deprivation injury in vitro^31,33^. Lactate is thus emerging as a potential neuroprotective agent^44^. In vivo studies have shown that the existence of a blood‒brain barrier makes it difficult for peripheral lactate to enter the brain, so intracerebral extracellular lactate is independent of blood lactate^45^. During hypoxia and exercise, the increase in brain lactate levels is less affected by the change in blood lactate levels. In 1998, Pellerin et al.^46^ found a distinct distribution of lactate between neurons and glial cells, suggesting that brain lactate is derived from astrocytes, while neurons serve as sites for lactate uptake and utilization. Lactate accumulation in the brain can not only cause delayed neuronal injury^21^ but can also act as an energy substrate for neuronal activity under certain circumstances^31^. In this study, pathological evaluation of neuronal death and determination of the brain lactate content were specifically designed to further investigate the mechanism of lactate damage and energy supply under exercise-induced fatigue. The results showed that with the extension of altitude acclimatization, the exercise capacity of rats gradually recovered, the brain lactate content of rats gradually increased, and the delayed neuronal injury and death were gradually alleviated. This recovery process was significantly blocked by 4-CIN, a lactate transport inhibitor. Based on the comprehensive analysis, we believe that brain lactate accumulation caused obvious neuronal injury after rush entry into altitude. MCT expression increases with the prolongation of altitude acclimatization. Then, brain lactate produced by glial cells can be taken up in part by neurons and used as an energy substrate in exercise-induced fatigue status, thus alleviating the neuronal injury caused by lactate accumulation, and the exercise capacity is gradually recovered. This protective effect was abolished when lactate transport was blocked by 4-CIN. We also believe that lactate accumulation is a relatively slow process. It can be seen that the increasing trend of the lactate content in rat brain in this study was gradually slowed down with the extension of altitude acclimatization, which is considered to be related to the utilization of part of the lactate by neurons for energy metabolism. We speculated that the expression of MCTs, the brain lactate content and the delayed neuronal injury would enter a stable state with the extension of altitude acclimatization. The above results fully illustrate the speculated mechanism involving the balance between brain lactate injury and energy supply in the state of exercise fatigue after altitude acclimatization.

Although only the central effects of fatigue were investigated in animal experiments in this study, the existence of a blood‒brain barrier makes it difficult for peripheral lactate to enter the brain, so intracerebral extracellular lactate is independent of blood lactate. The results have certain guiding significance for medical interventions for exercise-induced fatigue in high-altitude hypoxic environments. In further studies, siRNA interference and gene silencing should be used to artificially regulate MCT expression, which will provide more powerful evidence for this mechanism. Fatigue is an interactive effect resulting from both peripheral muscles and central factors, and the effect of high altitude on aerobic and anaerobic exercise capacity is different^47^. Submaximal exercise performance in humans improves with acclimatization primarily associated with improved oxygen delivery to exercising muscles, while maximal aerobic performance does not benefit much from acclimatization^48^. The changes in metabolic indices caused by fatigue are not limited to lactate. In addition, in vitro studies have shown that MCTs and excitatory amino acid transporters are involved in the protective mechanism of delayed neuronal injury^49,50^. All of the above are worthy of further exploration in altitude sports medicine research.

## Materials and methods

Reagents were obtained from Sigma‒Aldrich Corporation (Sigma‒Aldrich, St. Louis, MO, USA) except where otherwise noted.

### Experimental animals and groupings

This study was approved by the Animal Care and Use Committee of the 940th Hospital of Joint Logistics Support Force of Chinese People’s Liberation Army (Lanzhou, China) and followed the National Guidelines for Animal Experimentation. Experiments were performed on 63 male Sprague–Dawley rats (8 weeks old) weighing 280 ± 10 g that were obtained from the experimental animal centre at the hospital. Animals were individually housed in a room maintained at 18–24 °C, and the relative humidity of the air was 40–60% under a 12:12-h light/dark cycle, with access to food and water ad libitum. Experimental rats were randomly divided into seven groups (n = 9 in each group): a control group that was fed under normal pressure and normoxic conditions without incremental load exercise; a normal exercise group (NE group) that was fed under normal pressure and normoxic conditions for 2 weeks and then subjected to incremental load exercise; a rush entry into altitude group (REIA group) that was fed under normal pressure and normoxic conditions for 2 weeks and then subjected to incremental load exercise immediately under simulated high-altitude, low-pressure and hypoxic conditions without altitude acclimatization; a 3-day-altitude acclimatization group (3 d AA group) that was fed under normal pressure and normoxic conditions for 11 days and then transferred to simulated high-altitude, low-pressure and hypoxic conditions for 3 days and then subjected to incremental load exercise; a 1-week altitude acclimatization group (1 week AA group) that was successively fed under normal pressure and normoxic conditions or simulated high-altitude, low-pressure and hypoxic conditions for 1 week and then subjected to incremental load exercise; a 2-weeks altitude acclimatization group (2 weeks AA group) that was fed under simulated high-altitude, low-pressure and hypoxic conditions for 2 weeks and then subjected to incremental load exercise; and a monocarboxylate transporter inhibiter group (MTI group) that was fed under simulated high-altitude, low-pressure and hypoxic conditions for 2 weeks and then injected with monocarboxylate transporter inhibiter before exposing to incremental load exercise. Except for the control group, rats in the other groups were sacrificed after the end of the incremental load exercise without delay.

Except for the process of rat brain section preparation, animals were decapitated after anesthetized with isoflurane.

### Simulated high-altitude, low-pressure and hypoxic conditions

High-altitude, low-pressure and hypoxic conditions approximately 5000 m above sea level were simulated in the low-pressure and hypoxic animal experiment module (Model: FLYDWC50-IIA; Avic Guizhou Wind Thunder Aviation Ordnance Co. Ltd. Guizhou, China). The specific parameters were as follows: pressure 405.03 mmHg, partial pressure of oxygen 84.76 mmHg, temperature 12 °C (8:00 to 20:00 every day) and 2 °C (20:00 to 8:00 the next day), and relative humidity 30–40%.

### Exercise-induced fatigue model construction and inhibitor injection

The incremental load exercise protocol of Bedford^51^ was used to establish an exercise-induced fatigue model in rats. Except for the control group, rats in the other groups were subjected to training on the animal treadmill for 3 days of adaptation (speed: 15 m/min, time: 5 min/d, slope: 0°). After that, incremental load exercise was performed at a speed of 8.2 m/min × 15 min + 15 m/min × 15 min + 20 m/min until exhaustion. The criteria for exhaustion were as follows: The running gait of rats changed from a pedal ground type with the tail raised to a crawl ground type, called “belt riding”. Additionally, rats were stuck at the posterior one-third of the treadmill belt more than 3 times, failed to be driven by various stimuli, and exhibited shortness of breath, tiredness, and unresponsiveness after stopping running^52^.

A-cyano-4-hydroxycinnamate (4-CIN; 90 mg/kg), which is a specific covalent inhibitor of mitochondrial lactate and pyruvate transport^53^, was intraperitoneally injected 1 h before load exercise in the MTI group.

The exhaustive time of load exercise in rats was recorded and averaged in each group.

### Western blotting

Protein was extracted from the cerebral motor cortex of rats, and western blotting was performed as described previously^54^. After membrane transfer, the blots originating from different proteins were cut prior to hybridization with antibodies. Primary antibodies against MCT2 (Catalogue #: sc-50323, 1:1000; Santa Cruz Biotechnology Inc., CA, USA) and MCT4 (Catalogue #: PAB21410, 1:500; Abnova Corporation, Taipei City, Taiwan) were used, and a-tubulin (Catalogue #: 2144, 1:1,000; Cell Signaling Technology, Inc., Danvers, MA, USA) served as a loading control. Signals were detected using anti-mouse or anti-rabbit horseradish peroxidase-conjugated secondary antibodies (1:5,000; Abcam, Cambridge, MA, USA) and were analysed using ImageJ software (version number 1.38x/Java 1.6.0_02 https://imagej.nih.gov/ij/). The experiment was repeated three times and averaged.

### Rat brain section preparation and immunohistochemical staining methods (DAB method)

Rats were transcardially perfused with 0.1 M phosphate buffered saline (PBS, pH 7.4) and 4% paraformaldehyde in PBS after ansethetized with isoflurane. Then rats were decapitated and brains were removed and postfixed in paraformaldehyde (PFA) for 24 h at 4 °C and then dehydrated using graded sucrose phosphate buffer for 48 h. Coronal sections were cut on a freezing microtome at a thickness of 6 μm, and serial sections were collected consecutively in separate wells of an incubation chamber containing 0.1 M PBS with 60% glycerin.

Immunohistochemical detection was performed as described previously with some modifications^55^. Briefly, rat brain sections were blocked with 1% bovine serum protein (BSA) + 0.3% Triton X-100 for 1 h at room temperature, followed by washing with PBS. Then, the cells were incubated with antibodies against MCT2 (Catalogue #: sc-50323, 1:500; Santa Cruz Biotechnology, Inc. CA, USA) or MCT4 (Catalogue #: PAB21410, 1:200; Abnova Corporation, Taipei City, Taiwan) overnight at 4 °C on an orbital shaker and were washed. Biotin-labelled secondary antibody (reagent A) was added dropwise and incubated for 10 min at room temperature on an orbital shaker, followed by washing with PBS. Streptomycin antibiotic-peroxidase complex (reagent B) was dropped and incubated for 10 min at room temperature on an orbital shaker. After washing with PBS, the rat brain sections were transferred to slides, and freshly prepared DAB colour solution (20 times buffer 50 μl + 20 times substrate H_2_O_2_ 50 μl + 20 times DAB colour source 50 μl + deionized water 850 μl) was dropped onto the slides and developed in the dark. The primary antibody was replaced by antibody dilution as a blank control. After colour development, the slices were dehydrated, sealed with neutral gum, observed under a bright field microscope and photographed. The experiment was repeated three times and averaged.

### Neuropathological evaluation

Neuropathological evaluation of the hippocampus of rats in each group via Nissl staining with thionine (Solarbio, Beijing, China) was performed to determine the delay of neuron death by histological grade (HG) and neuronal density (ND)^56^.

Rat brain slices were dehydrated with alcohol, cleared with xylene and stained with thionine. Then, the thionine-stained brain tissues were subjected to HG and ND assessments. HG was divided into four grades and assigned as follows^57,58^: grade 0, no neuron death; grade I, scattered single neuron death; grade II, massive neuron death; and grade III, almost complete neuron death. ND was determined by counting the number of surviving pyramidal neurons with intact cell membranes, full nuclei, and clear nucleoli within a 250-μm linear length of the hippocampal CA1 subfield. The average number of pyramidal neurons in six random areas of the hippocampal CA1 subfield was calculated as the ND value.

### Determination of the lactate content in the rat brain

The brain tissue samples were weighed accurately and homogenized according to the weight (g):volume (ml) = 1:9 with normal saline to generate 10% brain tissue homogenates. After separation at 3,500 r/min for 10 min at 4 °C, the supernatant was loaded into EP tubes, and the lactate content was detected by a colorimetric method^59^. The operation was performed in strict accordance with the instructions of the kit.

### Statistical analysis

Statistical analyses were performed using SPSS v.16.0 for Windows (SPSS Inc., Chicago, IL, USA). All values are presented as the mean ± standard deviation (SD), and differences were determined using one-way ANOVA with Bonferroni or Tamhane’s T2 post hoc tests. P values < 0.05 were considered statistically significant.

The study protocol for each group is shown in Table 2.

**Table 2:** The study protocol for each group.

	Control	NE	REIA	3-d AA	1-week AA	2-week AA	MTI
Fed under normal conditions	Always	2 weeks	2 weeks	11 days	1 week	No	No
Fed under altitude conditions	No	No	No	3 days	1 week	2 weeks	2 weeks
Inhibitor intraperitoneal injection	No	No	No	No	No	No	Yes
Treadmill load exercise	No	Yes^#^	Yes*	Yes*	Yes*	Yes*	Yes*
Recorded exhaustive time^a^	No	Yes	Yes	Yes	Yes	Yes	Yes
Western blotting^b^	Yes	Yes	Yes	Yes	Yes	Yes	Yes
Immunohistochemical detection^c^	Yes	Yes	Yes	Yes	Yes	Yes	Yes
Neuropathological evaluation^c^	Yes	Yes	Yes	Yes	Yes	Yes	Yes
Brain lactate content determination^d^	Yes	Yes	Yes	Yes	Yes	Yes	Yes

Sample size: ^a^n = 9 per group; ^b^n = 3 per group; ^c^n = 3 per group; ^d^n = 3 per group.

Treadmill load exercise: *exercise under simulated high-altitude, low-pressure and hypoxic conditions; ^#^exercise under normal pressure and normoxic conditions.

### Medical ethics declarations and approval for animal experiments

All experimental animal protocols in this study were reviewed and approved by the Experimental Animal Ethics Committee of the 940th Hospital of Joint Logistics Support Force of Chinese People’s Liberation Army (Approval Letter 2020KYLL032), and all experimental protocols were carried out following guidelines such as the Animal Management Regulations (01/03/2017), Laboratory Animal: Guideline for Ethical Review of Animal Welfare (GB/T 35892-2018), ARRIVE 2.0, IGP 2012 and IAVE 2010.

## Supplementary Information

Supplementary Information.

## Data Availability

The datasets used and/or analysed during the current study are available from the corresponding author on reasonable request.

## Abbreviations

CNS: Central nervous system
MCTs: Monocarboxylate transporters
CP: Critical power
4-CIN: α-Cyano-4-hydroxycinnamate
PBS: Phosphate buffered saline
PFA: Paraformaldehyde
BSA: Bovine serum albumin
HG: Histological grade
ND: Neuronal density
SD: Standard deviation
