# Supplementary material for: A monocarboxylate transporter-dependent mechanism confers resistance to exercise-induced fatigue in a high-altitude hypoxic environment
Source: Sci Rep. 2023 Feb 20;13:2949. doi: 10.1038/s41598-023-30093-1 (PMC9941081; doi:10.1038/s41598-023-30093-1)

supplementary figure 1

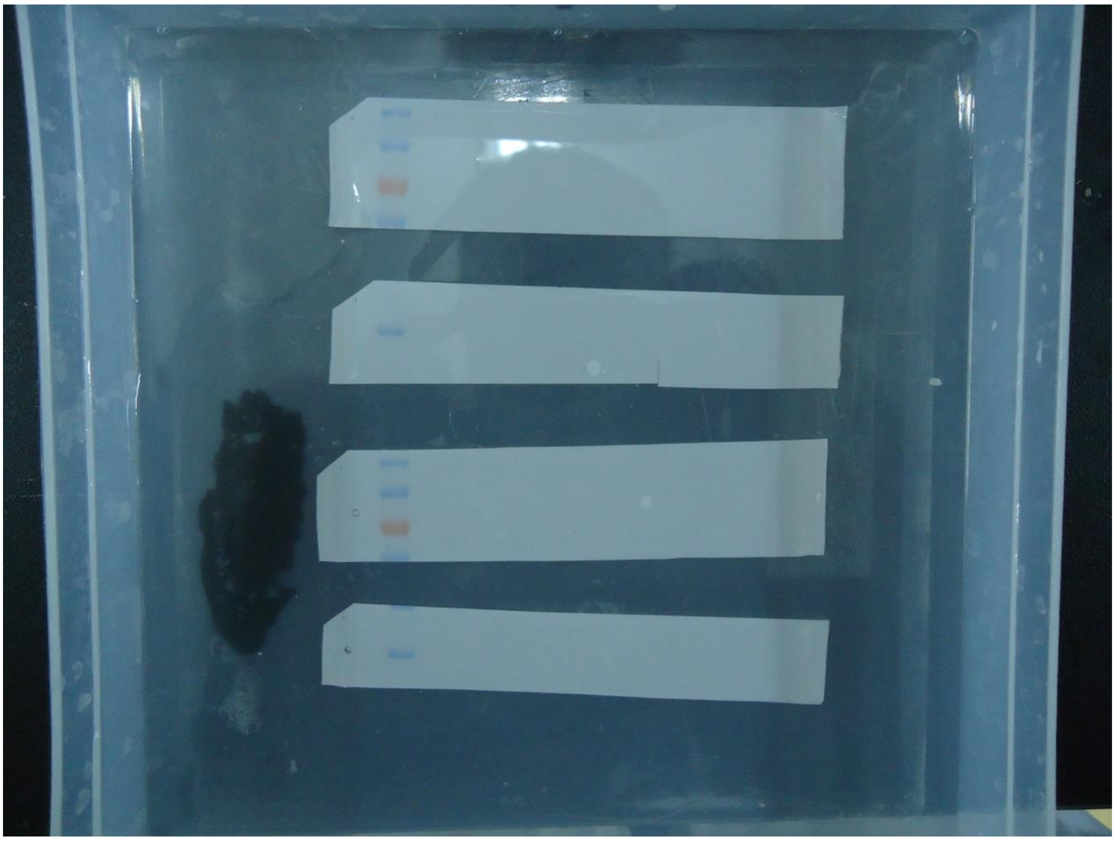

supplementary figure 2A

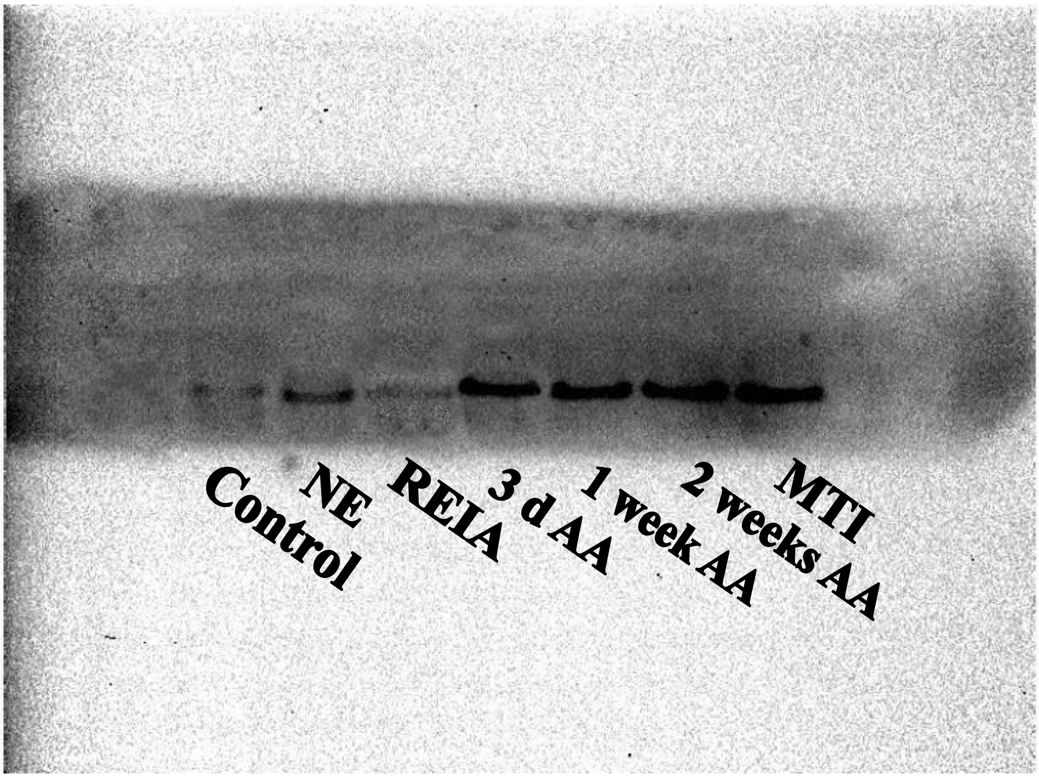

supplementary figure 2B

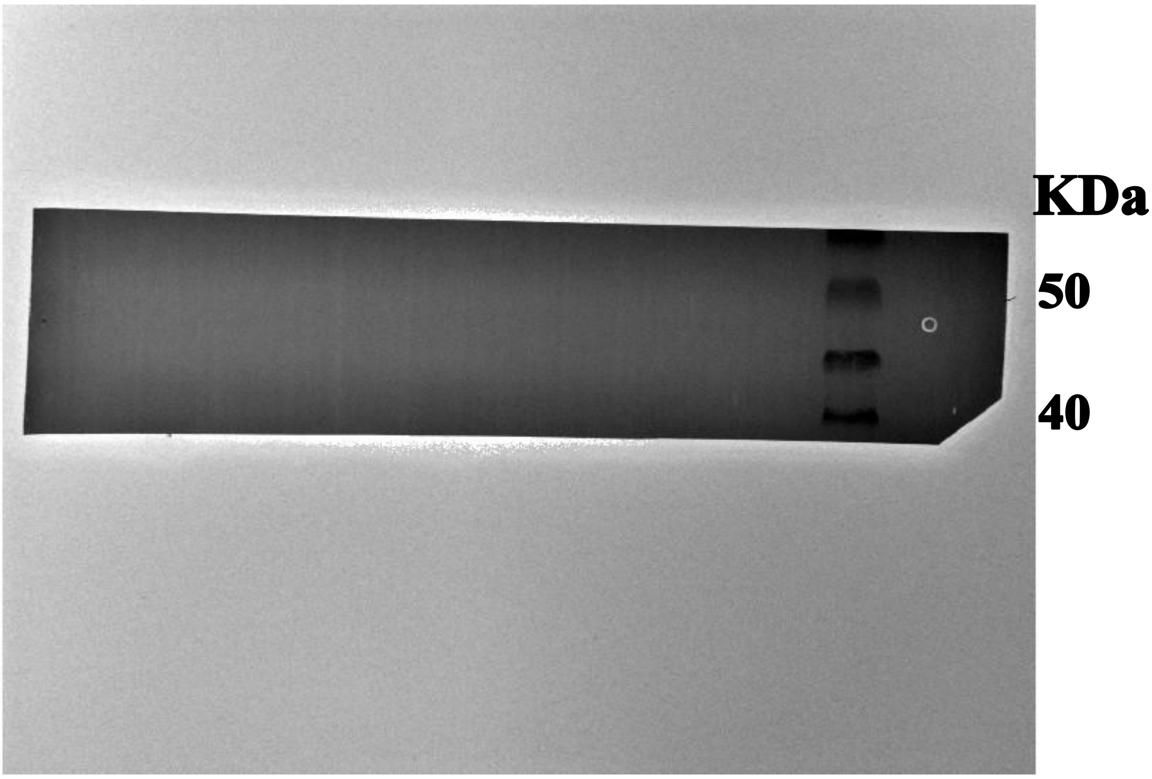

supplementary figure 3A

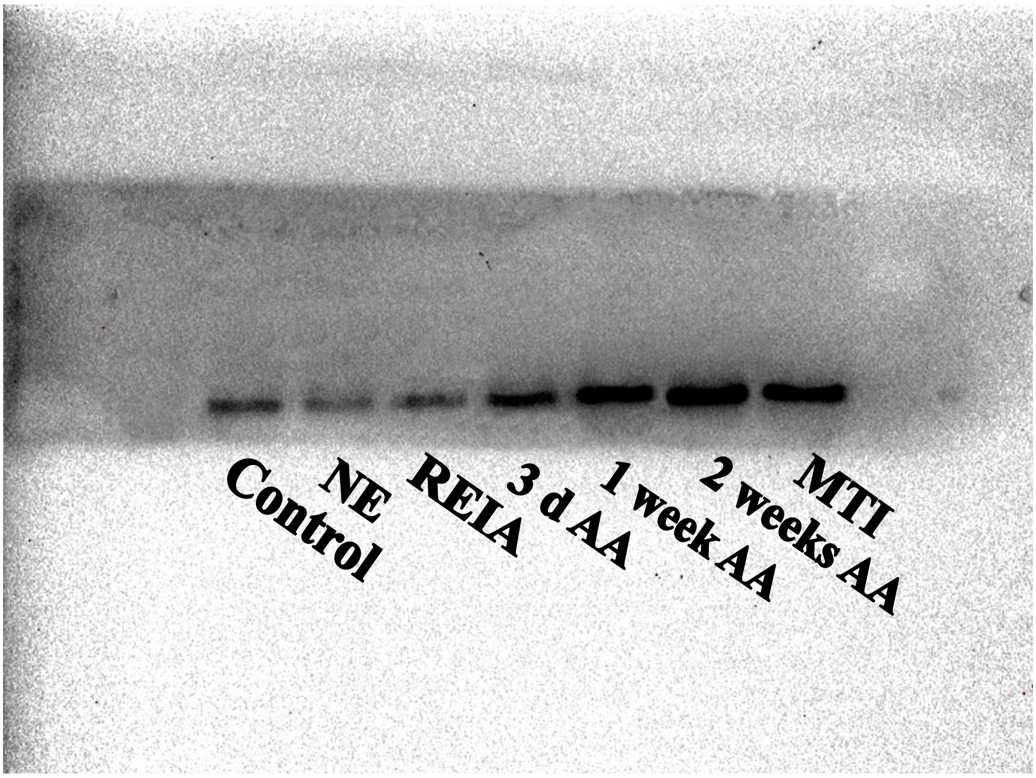

supplementary figure 3B

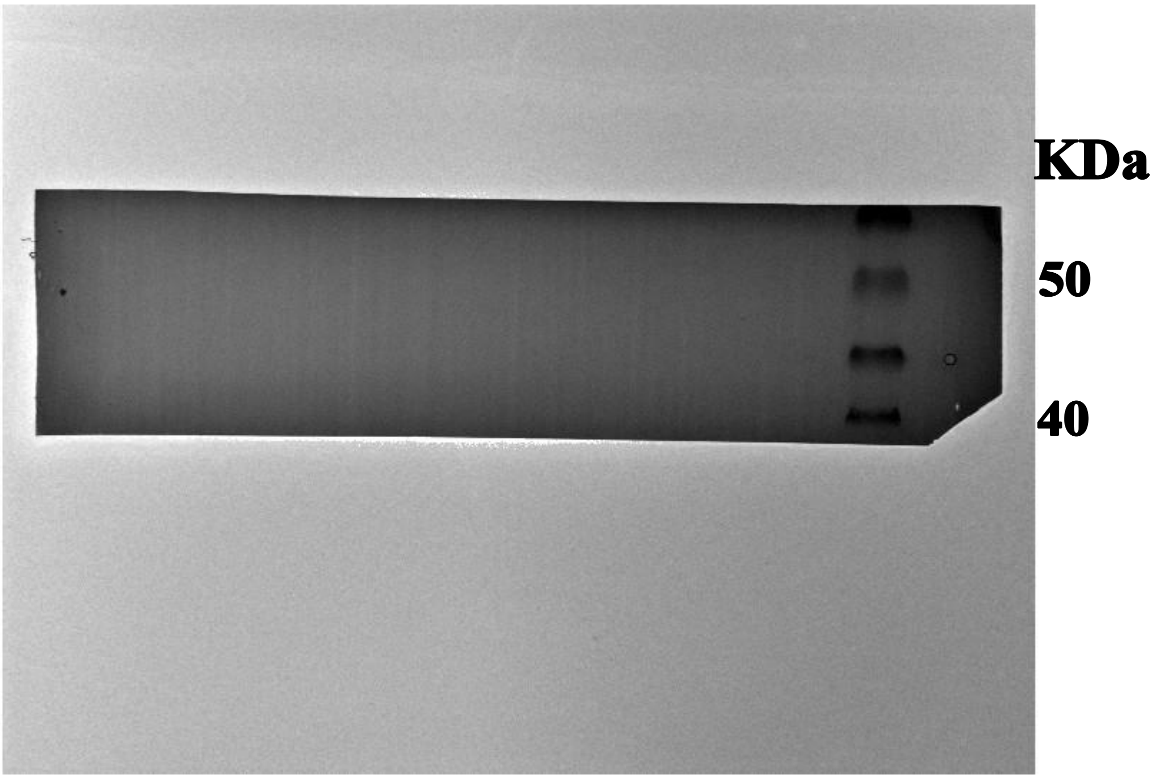

supplementary figure 4A

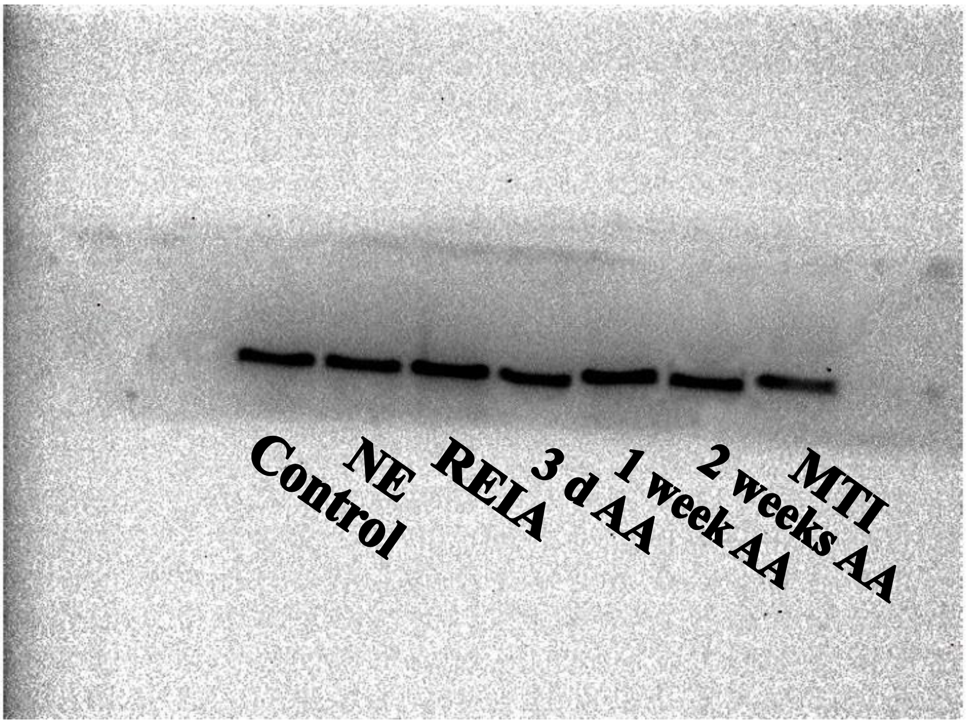

supplementary figure 4B

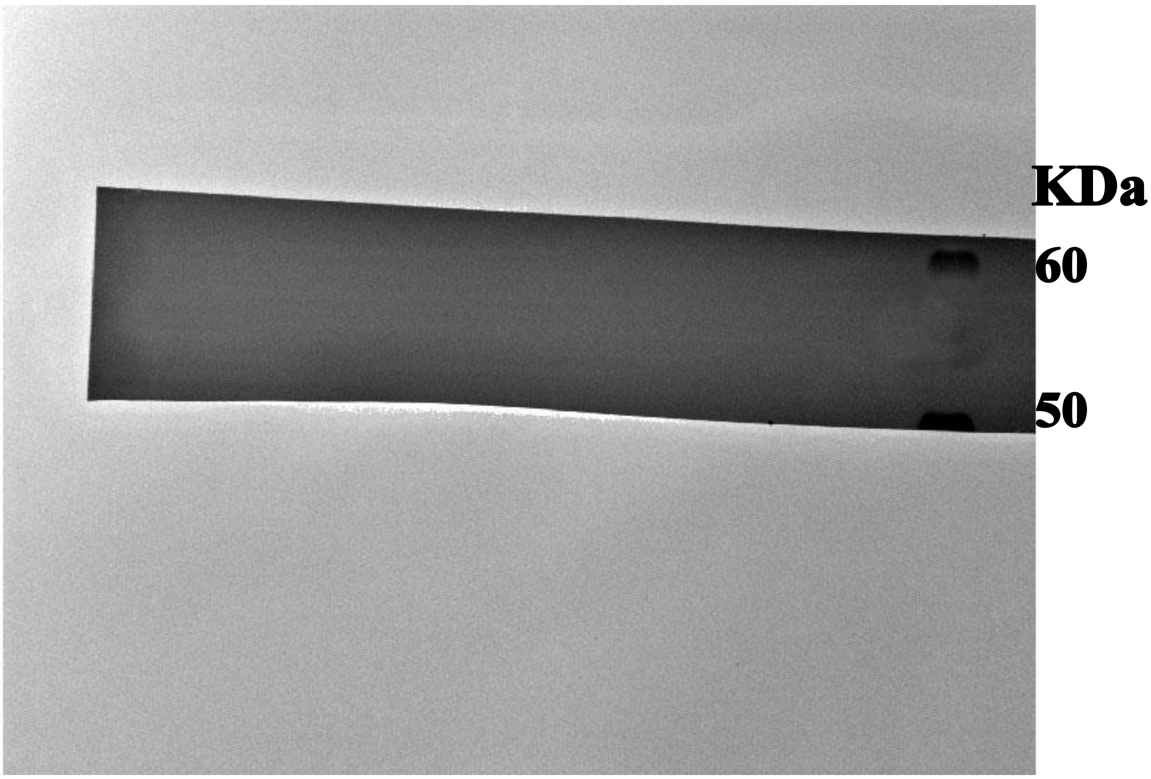

Supplement: Supplementary file 1 — Supplementary Information. [file 41598_2023_30093_MOESM1_ESM.pdf]
